# Loss of angulin-1/LSR promotes vasculogenic mimicry and epithelial-mesenchymal transition in breast cancer

**DOI:** 10.1016/j.jbc.2025.110635

**Published:** 2025-08-27

**Authors:** Yuma Yoshioka, Chisato Nosaka, Tomokazu Ohishi, Minami Nakajima, Yumiko Ishikawa, Tomoka Toyota, Masaru Takemae, Naoto Kubota, Jumpei Muramatsu, Daisuke Tatsuda, Masuo Kondoh, Hiroaki Onoe, Jiro Ando, Manabu Kawada, Hidenori Ojima, Siro Simizu

**Affiliations:** 1 Department of Applied Chemistry, Faculty of Science and Technology, Keio University, Yokohama, Japan; 2 Laboratory of Oncology, Institute of Microbial Chemistry (BIKAKEN), Microbial Chemistry Research Foundation, Shinagawa, Japan; 3 Division of Molecular Pathology, Research Institute, Tochigi Cancer Center, Utsunomiya, Japan; 4 Department of Breast Surgery, Tochigi Cancer Center, Utsunomiya, Japan; 5 Department of Mechanical Engineering, Faculty of Science and Technology, Keio University, Yokohama, Japan; 6 Graduate School of Pharmaceutical Sciences, Osaka University, Osaka, Japan; 7 Center for Infectious Disease Education and Research (CiDER), Osaka University, Osaka, Japan

**Keywords:** angulin-1/LSR, vasculogenic mimicry, breast cancer, cell junction, tight junction, EMT, biomarker

## Abstract

Vasculogenic mimicry (VM) is a process by which tumor cells form vessel-like network to secure oxygen and nutrients essential for tumor growth. Intercellular junctions, including tight junctions, may play a critical role in this process, important for VM. Here, we investigated the role of angulin-1/LSR (Ang-1), a key component of tricellular tight junctions, in VM regulation. We performed VM assay using Ang-1 KO breast cancer cells. Compared with control cells, Ang-1/KO cells exhibited significantly enhanced VM formation, accompanied by increasing epithelial-mesenchymal transition features. In a xenograft model, tumors derived from Ang-1/KO cells showed increased mass and elevated VM ratios. Re-expression of Ang-1 isoforms 3, 4, or 6-2 in Ang-1/KO cells suppressed VM formation, whereas isoforms 1, 2, and 6 had no effect. RT-PCR analysis of breast cancer patient tissues confirmed the lower expression of Ang-1 isoform 6-2, which suppresses VM. These findings identify Ang-1 as a novel regulator of VM and suggest that specific isoforms, particularly isoform 6-2, may serve as isoform-specific diagnostic markers or therapeutic targets in breast cancer.

The tight junctions (TJs) are essential components of intercellular adhesion that regulate epithelial and endothelial barrier functions and are increasingly recognized as important players in cancer progression (1). In breast cancer (BC), dysfunction of TJ proteins has been linked to enhanced proliferation, differentiation, and metastasis. TJs are composed of bicellular TJ (bTJ), located between two adjacent cells and tricellular TJ (tTJ), and formed at the contact point of three cells. Several studies have implicated bTJ proteins such as claudin-16 and occludin in tumor aggressiveness and suppression of tumorigenicity (2, 3), respectively, highlighting their functional importance in BC malignancy.

Angulin-1 (Ang-1), also known as lipolysis-stimulated lipoprotein receptor, is a critical component of tTJ and plays a key role in maintaining epithelial barrier integrity. Its cytoplasmic domain (CD) recruits tricellulin, a tTJ-specific protein, to the central sealing elements of tricellular contacts (4). Proper localization of Ang-1 is dependent on palmitoylated cysteine-rich (CR) domains in Ang-1 (5). Although multiple Ang-1 isoforms have been identified in cancer cells, the isoform-specific functions remain largely unexplored. Previous reports suggest a tumor-suppressive role for Ang-1, as its loss promotes cell invasion and migration in endometrial cancer *via* YAP signaling (6), and contributes to pancreatic cancer progression through growth factor pathways such as EGF (7).

Vasculogenic mimicry (VM) refers to the ability of aggressive cancer cells to form vessel-like networks independent of endothelial cells, thereby securing nutrition and oxygen supply (8). VM has been observed in multiple cancer types, including breast, lung, and prostate cancers, and is associated with poor prognosis (9, 10). Recent studies have suggested that integrin-β1–mediated cell–extracellular matrix interactions are critical for VM (11), while cell–cell junctions, especially those involving bTJ proteins such as claudin-4, also contribute to VM regulation (12). However, the involvement of tTJ proteins such as Ang-1 in VM remains unexplored.

In this study, we investigated the role of Ang-1 in VM by generating Ang-1 KO BC cells and evaluating VM capacity *in vitro* and *in vivo*. We found that loss of Ang-1 significantly enhances VM formation in T47D and MCF-7 cells, identifying Ang-1 as a negative regulator of VM. Re-expression of several Ang-1 isoforms revealed that only a subset suppresses VM, suggesting isoform-specific functionality. Furthermore, expression analysis of human BC patient samples confirmed the reduced expression of an inhibitory Ang-1 isoform 6-2. These findings establish Ang-1 as a novel isoform-dependent negative regulator of VM and implicate specific Ang-1 isoforms as potential diagnostic markers or therapeutic targets in BC.

## Results

### KO of Ang-1 promotes VM in BC cells

**Figure 1:**
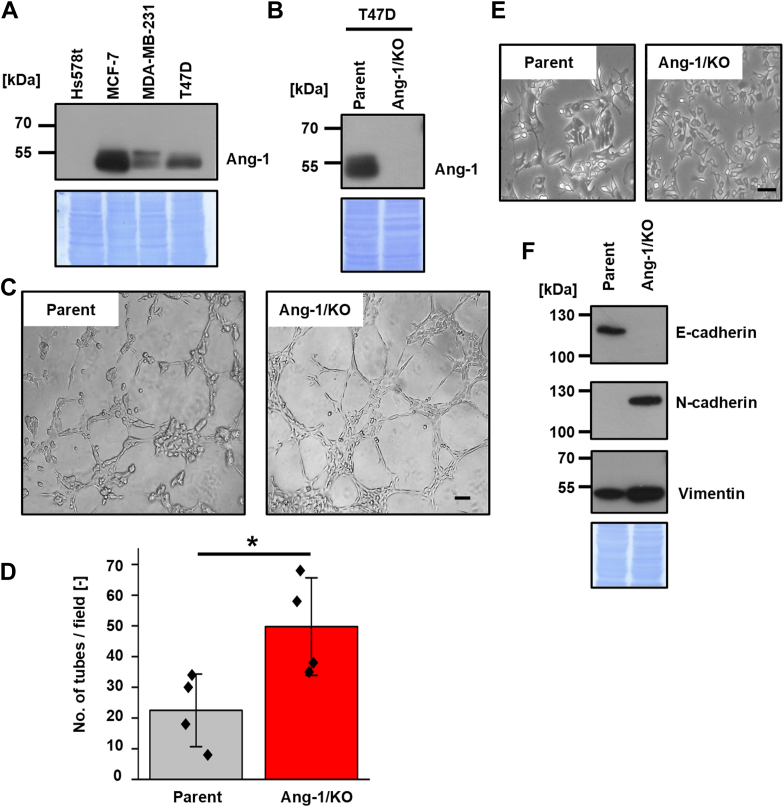
**KO of Ang-1 promotes VM in T47D cells.***A*, total lysates from four human breast cancer cell lines were subjected to SDS-PAGE and immunoblotted with anti-Ang-1 antibody. *B*, Ang-1/KO T47D cells were generated using the CRISPR/Cas9 system. Loss of Ang-1 expression was confirmed by immunoblotting. *C*, Ang-1/KO T47D cells were seeded on Matrigel-coated 96-well plates (1.6 × 10^4^ cells/well), and images were captured 5 h after seeding. *D*, quantification of tubular structures in four randomly selected fields (n = 3). *E*, representative images of parental and Ang-1/KO T47D cells cultured on Matrigel. *F*, expression levels of E-cadherin, N-cadherin and vimentin were assessed by Western blotting. CBB-stained membranes were used as loading controls. Data are presented as mean ± SD. ∗*p* < 0.05. Scale bars represent 100 μm. Ang-1, angulin-1; VM, vasculogenic mimicry.

**Figure 2:**
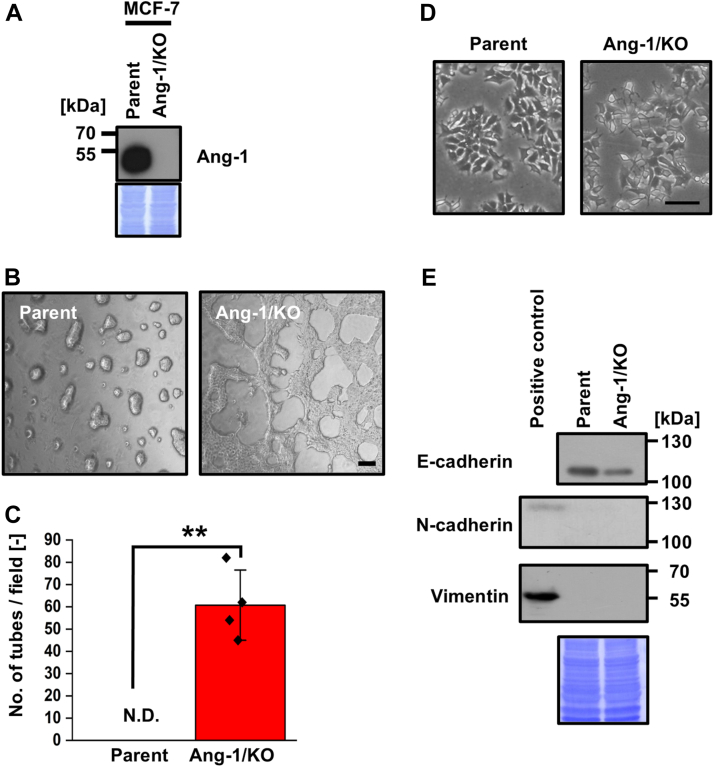
**KO of Ang-1 promotes VM in MCF-7 cells.***A*, Ang-1/KO MCF-7 cells were generated using the CRISPR/Cas9 system. Loss of Ang-1 expression was confirmed by immunoblotting. *B*, Ang-1/KO MCF-7 cells were seeded on Matrigel-coated 96-well plates (3.2 × 10^4^ cells/well), and images were captured 24 h after seeding. *C*, quantification of tubular structures in four randomly selected fields (n = 3). *D*, representative images of parental and Ang-1/KO MCF-7 cells cultured on Matrigel. *E*, expression levels of E-cadherin, N-cadherin, and vimentin were assessed by Western blotting. SK-MEL-28 cell lysate was used as positive control (N-cadherin- and vimentin-expressing cells). CBB-stained membranes were used as loading controls. Data are presented as mean ± SD. ∗∗*p* < 0.01. Scale bars represent 100 μm. Ang-1, angulin-1; VM, vasculogenic mimicry.

We performed immunoblot analysis to evaluate the expression of Ang-1 in various human BC cell lines. As shown in Figure 1*A*, Ang-1 was detected in MCF-7, T47D, and MDA-MB-231 cells, but not in Hs578t cells. Notably previous studies have demonstrated that the VM potential is highest in Hs578t cells, followed by MDA-MB-231, T47D, and MCF-7 cells (12, 13, 14), suggesting a possible inverse correlation between Ang-1 expression and VM capacity. To investigate this hypothesis, we established *Ang-1* KO in T47D and MCF-7 cells using CRISPR-Cas9 genome editing (Figs. 1*B* and 2*A*). Deletion of Ang-1 markedly enhanced VM formation in both cell lines compared with control cells (Figs. 1, *C* and *D* and 2, *B* and *C*), supporting the notion that Ang-1 negatively regulates VM.

To elucidate the mechanism by which Ang-1 loss promotes VM, we examined epithelial-mesenchymal transition (EMT), a process previously implicated in VM regulation (15). Morphological assessment revealed a mesenchymal-like phenotype in Ang-1/KO T47D cells relative to parental cells (Fig. 1*E*). Consistently, the expression of the epithelial marker E-cadherin was reduced, whereas the mesenchymal markers N-cadherin and vimentin were upregulated (Fig. 1*F*). In MCF-7 cells, Ang-1 deletion similarly reduced E-cadherin expression; however, no significant changes were observed in N-cadherin or vimentin levels (Fig. 2, *D* and *E*), suggesting a partial EMT phenotype. This result implied partial EMT in Ang-1/KO MCF-7 cells. Collectively, these findings indicate that Ang-1 suppresses VM in BC cells, at least in part, by modulating EMT-related pathways.

### Ang-1 KO enhances VM formation in xenograft tumor

**Figure 3:**
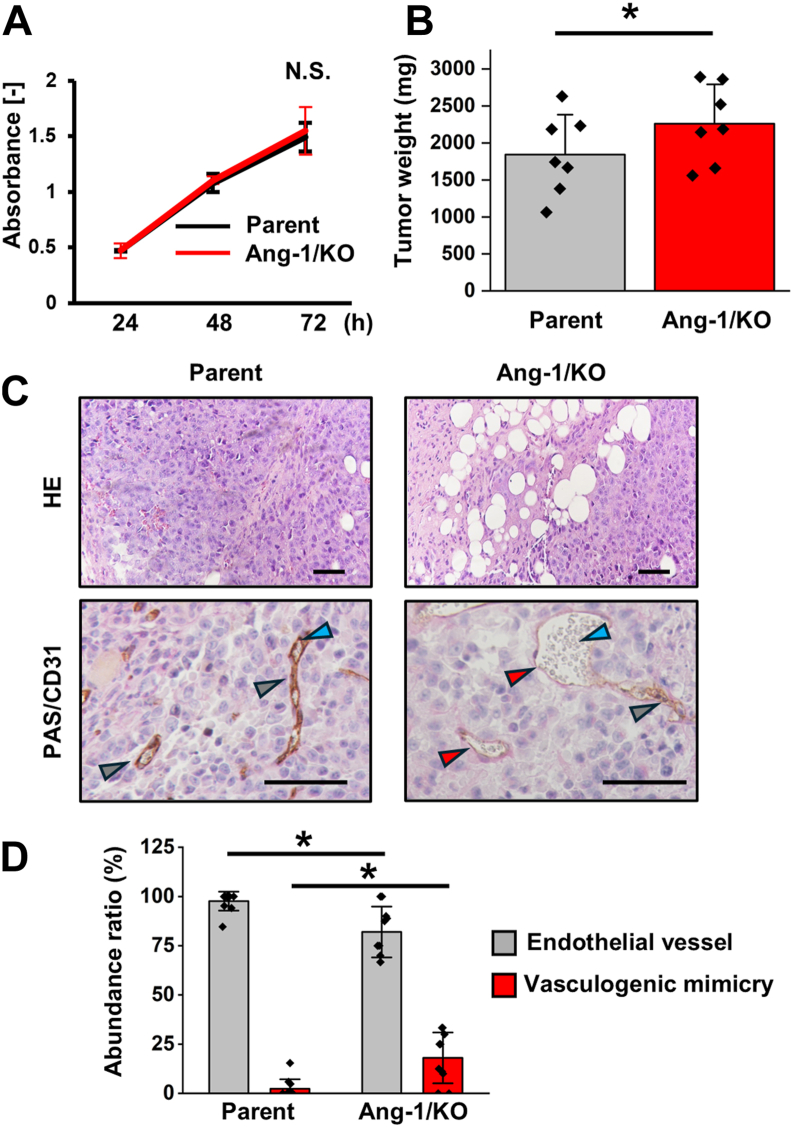
**Tumor weight is increased in Ang-1/KO xenograft tumors *via* enhancement of VM.***A*, parental T47D (*black*) and Ang-1/KO T47D (*red*) cells were seeded in 96-well plates (2000 cells/well) and cultured for 72 h. Cell proliferation was assessed by MTT assay every 24 h. *B*–*D*, parental and Ang-1/KO T47D cells (2.6 × 10^7^ cells/ml in serum-free DMEM) were mixed with Matrigel (final volume 0.8 ml), and 100 μl of the mixture was injected subcutaneously into the right and left groins of BALB/c nude mice, respectively (n = 7). Mice were sacrificed on day 14, and tumors were excised. *B*, tumor weights were measured, followed by fixation in 4% paraformaldehyde (pH 7.2) and paraffin embedding. *C*, representative histological images of H&E staining and periodic acid Schiff (PAS)/CD31 dual staining. *Red arrowheads* indicate VM channels (PAS-positive, CD31-negative), *gray arrowheads* indicate angiogenic vessels (PAS-positive, CD31-positive), and *blue arrowheads* indicate blood cells. *D*, the numbers of VM channels and angiogenic vessels were quantified by analyzing more than 10 representative microscopic fields, and the abundance ratio was calculated. Data are presented as mean ± SD. ∗*p* < 0.05. N.S., not significant. Scale bars represent 100 μm. Ang-1, angulin-1; DMEM, Dulbecco's modified Eagle's medium; MTT, 3-[4,5-dimethylthiazyol-2yl]-2,5-diphenyltetrazolium bromide; VM, vasculogenic mimicry.

To evaluate the impact of Ang-1 KO on VM *in vivo*, we first compared the proliferation of Ang-1/KO T47D cells with that of T47D parental cells *in vitro*. No significant difference in cell proliferation was observed between the two groups (Fig. 3*A*). We next established a xenograft model using parental and Ang-1/KO T47D cells. Tumors derived from Ang-1/KO cells exhibited significantly greater mass than those from parental cells (Figs. 3*B* and S1). VM was defined as periodic acid Schiff (PAS)-positive (PAS^+^) and CD31-negative (CD31^-^) vessels-like structures, as previously described (16, 17). In addition, structures containing blood cells—whether VM or angiogenic vessels—were considered functional. Histological analysis revealed a significant increase in the proportion of VM structures relative to the total numbers of vessels in Ang-1/KO tumors (Fig. 3, *C* and *D*). These results indicate that Ang-1 deletion promotes VM formation *in vivo*, thereby contributing to tumor growth independent of intrinsic proliferative capacity.

### Ang-1 isoforms 3, 4, and 6-2, but not 1, 2, and 6, suppress VM

**Figure 4:**
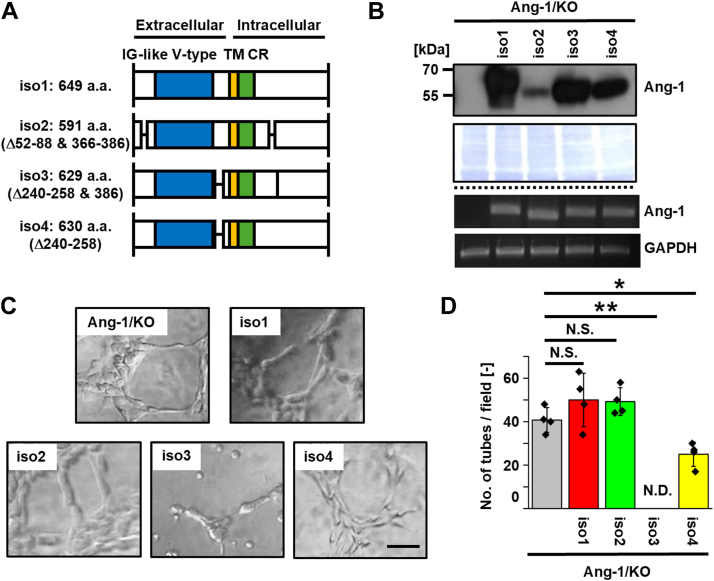
**Re-expressions of Ang-1 isoforms 1, 2, 3, and 4 reveal isoform-specific suppression of VM in T47D cells.***A*, schematic representation of the domain architecture of human Ang-1 isoforms 1, 2, 3, and 4 (designated as iso1–iso4; UniProt accession number Q86X29). Immunoglobulin (IG)-like V-type domain, transmembrane (TM) domain and cysteine-rich (CR) region are indicated by *blue*, *yellow*, and *green boxes*, respectively. *B*, T47D cells re-expressing individual Ang-1 isoforms were established. Protein expression was confirmed by immunoblotting, and mRNA expression of each isoform was verified by RT-PCR. *C*, cells were seeded on Matrigel-coated 96-well plates (2.0 × 10^4^ cells/well), and images were captured 5 h after seeding. *D*, the number of tubular structures was quantified in four randomly selected fields (n = 3). Data are presented as mean ± SD. ∗*p* < 0.05, ∗∗*p* < 0.01. N.D., not detected; N.S., not significant. The scale bar represents 100 μm. Ang-1, angulin-1; VM, vasculogenic mimicry.

**Figure 5:**
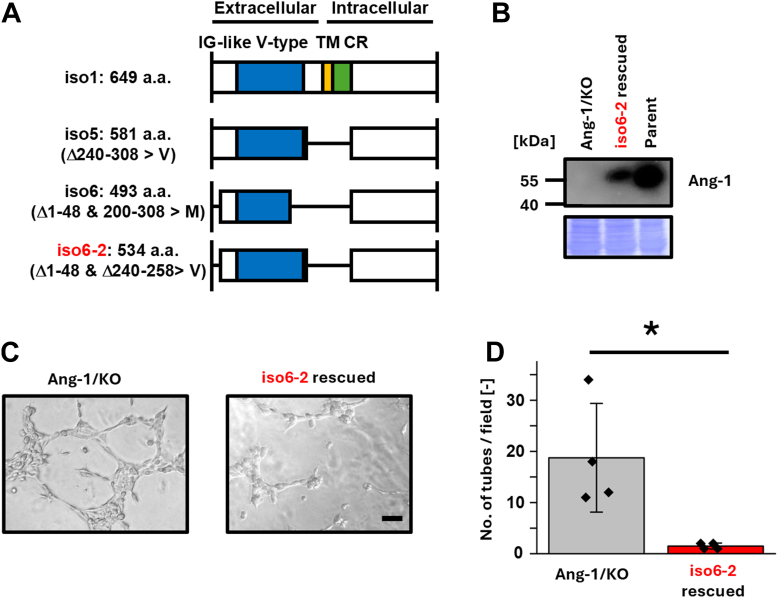
**Inhibitory effects of Ang-1 isoform 6-2 on VM in T47D cells.***A*, schematic representation of the domain architecture of human Ang-1 isoforms 5, 6, and 6-2 (designated as iso5, iso6, and iso6-2; UniProt accession number Q86X29). The immunoglobulin (IG)-like V-type domain is indicated by *blue box*. *B*, T47D cells re-expressing Ang-1 isoform 6-2 were established. Protein expression was confirmed by immunoblotting. *C*, cells were seeded on Matrigel-coated 96-well plates (2.0 × 10^4^ cells/well), and images were captured 5 h after seeding. *D*, the number of tubular structures was quantified in four randomly selected fields (n = 3). Data are presented as mean ± SD. ∗*p* < 0.05. The scale bar represents 100 μm. Ang-1, angulin-1; VM, vasculogenic mimicry.

According to the UniProt protein database, Ang-1 has several isoforms, including isoforms 1 to 4 (Fig. 4*A*). To assess these roles in VM, we re-expressed individual Ang-1 isoforms in T47D/KO cells (Fig. 4*B*) and evaluated VM formation. Isoforms 1 to 4 were selected based on the presence of both transmembrane (TM) and CR domains, which are absent in isoforms 5 and 6 (Fig. 5*A*). VM formation was significantly suppressed in isoform 3– and 4–expressing cells compared with Ang-1/KO cells (Fig. 4, *C* and *D*), whereas isoforms 1 and 2 had no appreciable effect.

**Figure 6:**
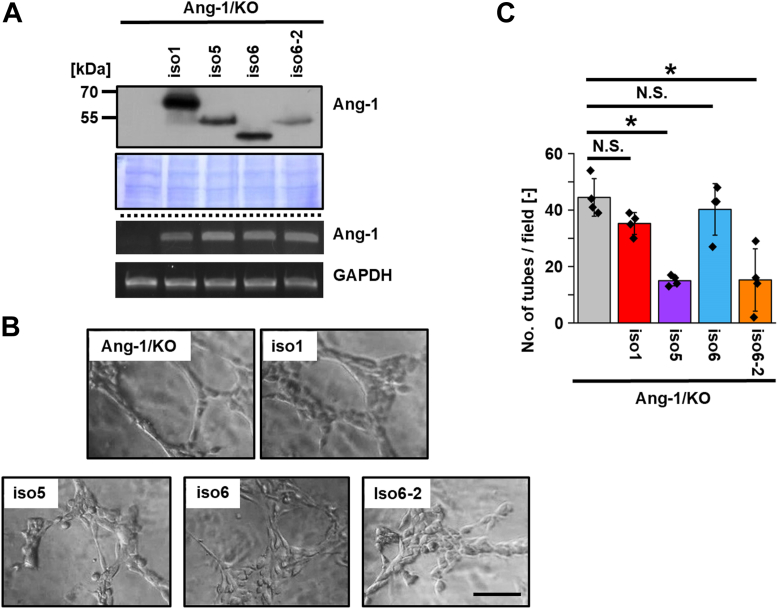
**Re-expressions of Ang-1 isoforms 1, 5, 6, and 6-2 reveal isoform-specific suppression of VM in T47D cells.***A*, T47D cells re-expressing Ang-1 isoforms 5, 6, and 6-2 were established. Protein expression was confirmed by immunoblotting, and mRNA expression of each isoform was verified by RT-PCR. *B*, cells were seeded on Matrigel-coated 96-well plates (2.0 × 10^4^ cells/well), and images were captured 5 h after seeding. *C*, the number of tubular structures was quantified in four randomly selected fields (n = 3). Data are presented as mean ± SD. ∗*p* < 0.05. The scale bar represents 100 μm. Ang-1, angulin-1; VM, vasculogenic mimicry.

**Figure 7:**
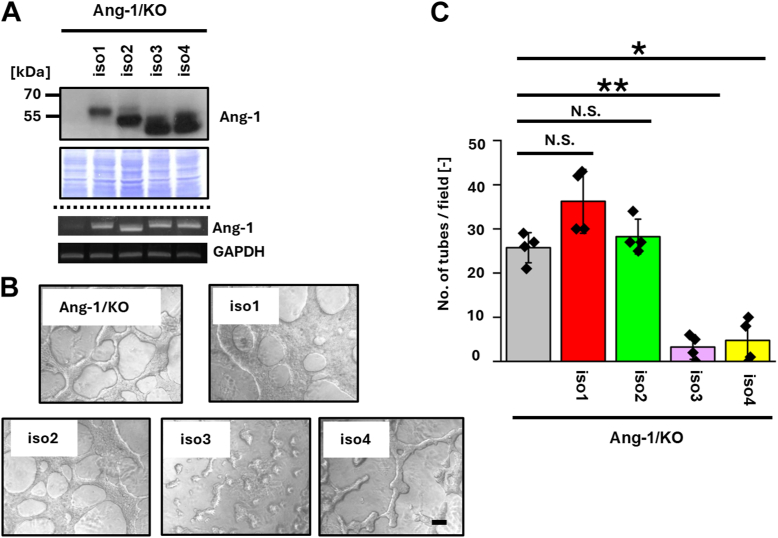
**Re-expressions of Ang-1 isoforms 1, 2, 3, and 4 reveal isoform-specific suppression of VM in MCF-7 cells.***A*, MCF-7 cells re-expressing individual Ang-1 isoforms were established. Protein expression was confirmed by immunoblotting, and mRNA expression of each isoform was verified by RT-PCR. *B*, cells were seeded on Matrigel-coated 96-well plates (4.8 × 10^4^ cells/well), and images were captured 24 h after seeding. *C*, the number of tubular structures was quantified in four randomly selected fields (n = 3). Data are presented as mean ± SD. ∗*p* < 0.05, ∗∗*p* < 0.01. N.S., not significant. The Scale bar represents 100 μm. Ang-1, angulin-1; VM, vasculogenic mimicry.

**Figure 8:**
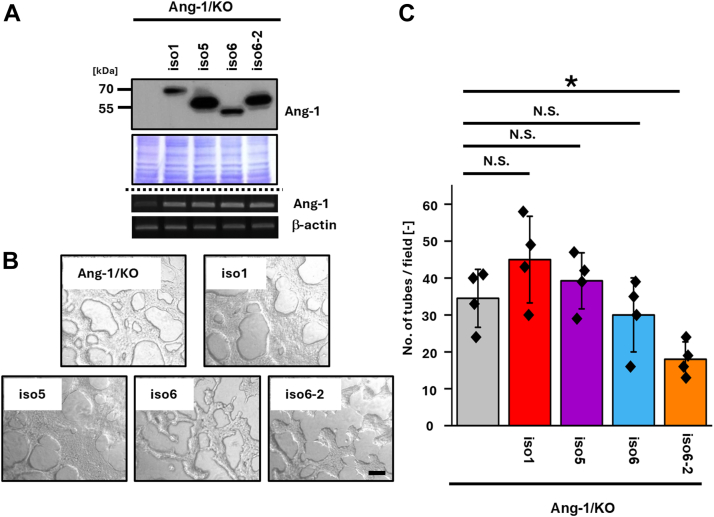
**Re-expressions of Ang-1 isoforms 1, 5, 6, and 6-2 reveal isoform-specific suppression of VM in MCF-7 cells.***A*, MCF-7 cells re-expressing Ang-1 isoforms 5, 6, and 6-2 were established. Protein expression was confirmed by immunoblotting, and mRNA expression of each isoform was verified by RT-PCR. *B*, cells were seeded on Matrigel-coated 96-well plates (4.8 × 10^4^ cells/well), and images were captured 24 h after seeding. *C*, the number of tubular structures was quantified in four randomly selected fields (n = 3). Data are presented as mean ± SD. ∗*p* < 0.05. The scale bar represents 100 μm. Ang-1, angulin-1; VM, vasculogenic mimicry.

To identify the endogenous Ang-1 isoform of Ang-1 in T47D cells, we cloned the Ang-1 cDNA and determined its amino acid sequence. The resulting product, designated isoform 6-2, lacked residues 240 to 308, which were replaced with valine and consequently omitted both the TM and CR domains (Fig. S2). Re-expression of isoform 6-2 in Ang-1/KO T47D cells suppressed VM formation (Fig. 5), consistent with the VM-inhibitory effects observed for isoforms 3 and 4. We further examined isoforms 5 and 6. Re-expression of isoform 5, but not isoform 6, suppressed VM in T47D cells (Fig. 6). To evaluate these findings in another model, we established MCF-7 cell lines stably expressing isoforms 1 to 6 and 6-2 (Figs. 7 and 8). Isoforms 3, 4, and 6-2 exhibited reduced VM formation in MCF-7 cells, consistent with results in T47D cells. In contrast, isoforms 1, 2, and 6 did not affect VM, and isoform 5 unexpectedly failed to suppress VM in MCF-7 cells, in contrast to its effect in T47D cells. Collectively, these results demonstrate that Ang-1 regulates VM in an isoform-specific and context-dependent manner.

### Ang-1 isoform 6-2 has a suppressive effect on cancer malignancy in BC patients

**Figure 9:**
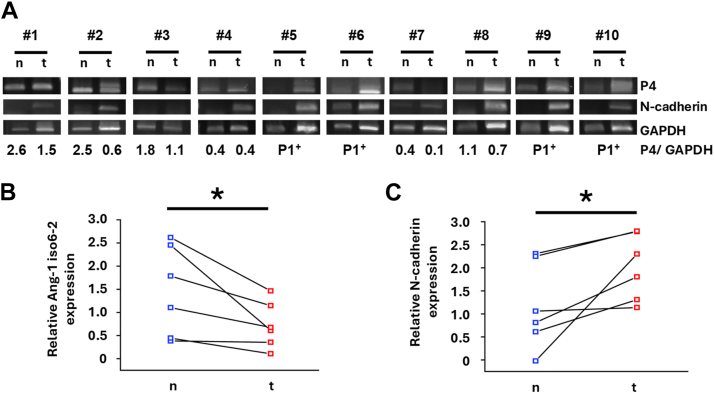
**Ang-1 isoform 6-2 has a suppressive role on cancer malignancy in BC patients.***A*, RT-PCR products of Ang-1 isoform 6-2, N-cadherin and GAPDH from 10 BC patients. *B* and *C*, slope graphs show the results of semiquantitative PCR for Ang-1 isoform 6-2 (P4; Fig. S3*A*), N-cadherin, and GAPDH. Both genes were normalized by GAPDH. ∗*p* < 0.05. P1^+^, patient-derived samples in which only isoform 6-2 expression could not be detected, due to amplification with the P1 primer set shown in Fig. S3. Ang-1, angulin-1; BC, breast cancer.

Since endogenous Ang-1 isoform 6-2 was detected in both T47D and MCF-7 cells (Fig. S2), we investigated whether Ang-1 isoform 6-2 also present in BC patients and whether its expression is reduced compared to matched normal tissues. To this end, we designed multiple primer sets and experimentally validated that each functioned intended (Fig. S3, *A* and *B*). RT-PCR analysis of four human BC cell lines confirmed the presence of Ang-1 isoform 6-2 existed in all cell lines, consistent with the results obtained from T47D cells (Fig. S3*C*). We then examined Ang-1 isoform 6-2 expresses in primary breast tumors and matched normal tissues from 10 BC patients using RT-PCR (Fig. 9*A*). Ang-1 isoform 6-2 was detected in a subset of tumor samples, while in others, isoforms distinct from Ang-1 isoforms 6 and 6-2 were observed. Among the cases expressing Ang-1 isoform 6-2, tumor tissues exhibited significantly lower levels of Ang-1 isoform 6-2 than their matched normal counterparts (Fig. 9*B*). Notably, this reduction in Ang-1 isoform 6-2 expression was associated with a significant upregulation of N-cadherin in tumor tissues relative to normal tissues (Fig. 9*C*). These findings suggest that reduced Ang-1 isoform 6-2 expression may contribute to BC development through the induction of partial EMT.

## Discussion

Although numerous antiangiogenic drugs have been developed for cancer therapy, their efficacy remains limited in certain patient populations (18). In BC, for example, the antiangiogenic agent sunitinib has been reported to paradoxically promote cancer invasion through VM (19). VM refers to a process in which cancer cells, rather than vascular endothelial cells, form vessel-like structures to supply oxygen and nutrients to tumors. While cell–cell adhesion among cancer cells has been considered important for VM, mechanistic studies are limited.

In this study, we focused on Ang-1, a tTJ protein involved in cell–cell junctions, and demonstrated that Ang-1 suppresses VM both *in vitro* and *in vivo* in an isoform-dependent manner. Specifically, expression of Ang-1 isoforms 3, 4, and 6-2 inhibited VM, whereas isoforms 1, 2, and 6 had no appreciable effect (Figure 4, Figure 5, Figure 6, Figure 7, Figure 8). We do not attribute the lack of VM suppression in Ang-1 isoform 2–expressing cells to insufficient mRNA expression, as mRNA levels were comparable among isoform-expressing cells. Instead, the protein level of Ang-1 isoform 2 was markedly reduced, suggesting that this isoform may be inherently unstable in T47D cells (Fig. 4*B*). Similar isoform-dependent VM-suppressive patterns were observed in MCF-7 cells (Fig. 7), supporting the notion that the limited effect of Ang-1 isoform 2 is not due to differential expression levels but rather to isoform-specific properties. Interestingly, Ang-1 isoform 5 inhibited VM formation in T47D cells but not in MCF-7 cells, suggesting that the functional consequences of Ang-1 isoforms may be cell line-specific.

Previous studies have shown that cysteine residues within the CR domain of Ang-1 are palmitoylated, a modification essential for its localization to tricellular contacts (5). Additionally, the CD contributes to Ang-1 trafficking and membrane retention. Despite isoform 1 being the canonical sequence in UniProt, it failed to suppress VM in our assays. To elucidate the underlying mechanism, we generated mutant forms of Ang-1 isoform 1: Ang-1/ΔCD, in which the CD (excluding the CR domain) was deleted, and Ang-1/CS, in which 10 putative palmitoylation sites in the CR domain were replaced with serine (Fig. S4*A*). Transfection of these constructs into Ang-1/KO MCF-7 cells revealed that both Ang-1/ΔCD and Ang-1/CS significantly reduced VM compared to full-length Ang-1/iso1 (Fig. S4, *C*–*E*), indicating that the CR domain and CD play a role in the regulation of VM.

Our analysis using various deletion mutants of Ang-1 (Figure 4, Figure 5, Figure 6, Figure 7, Figure 8 and S4) further suggests that the tTJ function of Ang-1 is not directly linked to its ability to suppress VM. For example, although isoforms 1 through 4 all contain a TM domain, they exhibit differing VM-suppressive capacities. To investigate whether disruption of tTJ function *per se* promotes VM, we treated T47D cells with angubindin-1, a peptide ligand known to inhibit Ang-1 function (20). While angubindin-1 downregulated Ang-1 expression, VM formation remained unaffected (data not shown).

We propose that the suppressive effects of isoforms 3, 4, and 6-2 on VM arise from distinct structural mechanisms. For instance, isoforms 3 and 4 retain TM and CR domains but carry specific deletions—residues 240 to 258 and 386, respectively—which may impair VM formation. In contrast, isoform 6-2, which lacks both the TM and CR domains, contains deletions of residues 1 to 48 and 240 to 308 that appear necessary for VM inhibition. These findings suggest that VM suppression by Ang-1 is governed by isoform-specific structural motifs rather than classical tTJ-related domains. Future studies will explore whether Ang-1 exerts similar effects on VM in other cancer types, such as colorectal cancer, and whether it modulates EMT in a broader oncogenic context.

Analysis of publicly available data using the Gene Expression Profiling Interactive Analysis database revealed no significant difference in overall survival between BC patients with high *versus* low Ang-1 expression (Fig. S5). This suggests that evaluating total Ang-1 expression, without distinguishing among isoforms, may be insufficient for prognostic assessment. In contrast, expression levels of Ang-1 isoforms 3, 4, and 6-2 may serve as more accurate prognostic markers in BC.

Notably, we identified Ang-1 isoform 6-2, a previously unannotated isoform absent from the UniProt database, as an endogenous variant expressed in T47D and MCF-7 cells, as well as in some BC patient tissues. This suggests that isoform 6-2, rather than isoform 1, may represent the physiologically predominant form of Ang-1 in BC, consistent with prior reports in colon carcinoma cell line (21). KO of Ang-1 isoform 6-2 enhanced VM and was associated with EMT induction (Figs. 1 and 2). *In vivo*, Ang-1/KO T47D xenografts exhibited increased VM and tumor mass relative to parental tumors (Fig. 3). Furthermore, Ang-1 isoform 6-2 expression was consistently lower in tumor tissues than matched normal tissues, and this downregulation was accompanied by upregulation of N-cadherin—an EMT marker—suggesting a partial EMT state. E-cadherin and vimentin levels were not significantly altered (Fig. S3*C*), supporting the hypothesis that partial EMT may underly the observed phenotypes. This is consistent with recent studies indicating that cancer cells rarely transition fully between epithelial and mesenchymal states (22, 23), instead occupying intermediate states that vary by cell type and context. Accordingly, we propose Ang-1 isoform 6-2 as a potential prognostic marker and functional regulator of VM and EMT in BC.

## Experimental procedures

### Cell culture

Human BC cells Hs578t were purchased from ATCC and MCF-7, MDA-MB-231, and T47D cell lines were obtained from RIKEN BioResource Research Center. Cells were cultured in AccuDia Dulbecco's modified Eagle's medium (DMEM; Shimadzu Diagnostics Co, Ltd) that was supplemented with 10% (v/v) fetal bovine serum, 100 U/ml penicillin G, 100 mg/l kanamycin, 600 mg/l L-glutamine, and 2.25 g/l NaHCO_3_ in a humidified incubator with 5% CO_2_ at 37 °C.

### VM assay

VM assay was performed as previously described (24, 25, 26). Briefly, 96-well plates were coated with 40 μl/well Matrigel growth factor reduced (Corning) and incubated for 30 min at 37 °C. The cells were seeded onto the Matrigel-coated wells and cultured at 37 °C in a humidified incubator with 5% CO_2_. The wells were photographed by phase-contrast microscope (Leica DMi1, Leica), and the number of tubes was counted in 4 random independent fields. We defined capillary-like tubes by VM as areas surrounded by cells. Since T47D and MCF-7 cells have different capacities for VM formation, the cells were seeded at different densities accordingly.

### CRISPR-mediated Ang-1 gene KO

Ang-1 gene was knocked out using the CRISPR-Cas9 system previously described (27, 28, 29). To reduce off-target effects, we used a D10A Cas9 nickase mutant with paired single guide RNAs. Paired single guide RNAs that targeted exon 2 of Ang-1 gene were generated using the following oligonucleotides: target 1, 5′-CACCGATTGAGCTGGTTGTCGACGC-3′ (forward) and 5′-AAACGCGTCGACAACCAGCTCAATC-3′ (reverse); target 2, 5′-CACCGCAGCTGGCAGCCGGGAACCC-3′ (forward) and 5′-AAACGGGTTCCCGGCTGCCAGCTGC-3′ (reverse). Guide oligos were inserted separately into the BbsI restriction site of the pSpCas9n(BB)-2A-Puro (PX462) V2.0 plasmid (Addgene), gifted from Prof. Feng Zhang.

Two vectors, each containing 1 guide RNA sequence, were cotransfected into cancer cells using Lipofectamine 3000 (Thermo Fisher Scientific), which were then treated with 2 μg/ml puromycin dihydrochloride (Merck KGaA) to select transfectants. Clonal Ang-1/KO cells were established by the limiting dilution method.

### Immunoblot

Western blot was performed as previously described (30, 31, 32, 33, 34). Cells were lysed with the buffer that contained 50 mM, Tris–HCl pH 7.5, 150 mM NaCl, 0.1% (w/v) SDS, 1% (v/v) Triton X-100, 1% (w/v) sodium deoxycholate, and 1 mM PMSF on ice with sonication. The lysate was centrifuged at 13,000*g* for 10 min, and the protein concentrations in each lysate were measured with Coomassie Brilliant Blue R-250 (Bio-Rad Laboratories) by Bradford method. Loading buffer [350 mM Tris–HCl pH 6.8, 30% (w/v) glycerol, 0.012% (w/v) bromophenol blue, 6% (w/v) SDS, and 30% (v/v) 2-mercaptoethanol] was added to each lysate and mixed samples were boiled for 3 min. The samples were electrophoresed on 10% SDS/polyacrylamide gels, after which the proteins were transferred to polyvinylidene fluoride membranes. Proteins were immunoblotted with anti–lipolysis-stimulated lipoprotein receptor (Sigma-Aldrich, HPA007270), anti-E-cadherin (Santa Cruz, SC-21791), anti-N-cadherin (Santa Cruz, sc-393933), anti-vimentin (Santa Cruz, sc-373717), and anti-GFP (Santa Cruz, sc-9996). The secondary antibodies were HRP-linked-anti-mouse IgG (Cytiva #NA931) and HRP-linked-anti-rabbit IgG (Cytiva #NA934). Signals were detected with Immobilon Western Chemiluminescent HRP substrate (Merck KGaA #WBKLS0500) and exposed to RX-U films (FUJIFILM) in the dark room. The membrane was stained with CBB.

### Plasmid construction

We thank the Research Association for Biotechnology, Dr Yoshihide Hayashizaki of RIKEN, and Dr Sumio Sugano of the University of Tokyo for providing the IRAK091D16 (cat. HGY036488) through the National BioResource Project of the MEXT. Full-length cDNA encoding human Ang-1 was amplified from IRAK091D16 (bought by RIKEN BioResource Research Center) by PCR with the following primers, 5′- TTTTGAATTCATGCAACAGGACGGACTTGG-3′ (forward) and 5′- TTTTGCGGCCGCTCAGACGACTAAACTTTCCC-3′ (reverse). Considering its potential usefulness in future experiments, we generated Cas9-resistant Ang-1 by inverse PCR with the following primers: 5′- CCCAACTCGCCGCTGGCAATCCCGGGTACAACCCCTACGTTGAGTG-3′ (forward) and 5′-TCGCCGATGCCTTCTCCCCGGCTAGTGTGGATAATCAACTGAACG-3′ (reverse). Ang-1 isoforms 2, 3, 4, and 5 were introduced by inverse PCR with the following primers: the former deletion part of isoform 2, 5′-GCCATCCAGGTGACCGTGTCCAAC-3’ (forward) and 5′-CAGCGCCATCGCGGCCGTC-3′ (reverse), the latter deletion part of isoform 2, 5′- GTCCGCAGTGGCTACAGGATTC-3′ (forward) and 5′-TGAGCTACTCCTGTCAACGTCTCC-3′ (reverse), isoform 3 (used as a template of isoform 4 plasmid), 5′-GTCCGCAGTGGCTACAGGATTC-3′ (forward), and 5′-AGAGGCCACACTGCTGTCCG-3′ (reverse), isoform 4, 5′-GACTGGCTCTTCGTGGTTGTG-3′ (forward) and 5′-AAGGACGATGAGCTCTGCGTAG-3′ (reverse) and isoform 5, 5′-TGTATGCCGCCGGCAAAGCAGCCACCTCAG-3′ (forward) and 5′- CAAGGACGATGAGCTCTGCGTAGGCCTCATTGTTC-3′ (reverse). Ang-1 isoform 6, were introduced by overlap extension techniques with the following primers: the former deletion part of iso6, 5′-TTTTGAATTCATGGCGCTGTTGGCCGGC-3′ (forward) and 5′-GCGGCATACATTCCGGTGATGGTAATCCTC-3’ (reverse), the latter deletion part of isoform 6, 5′-ATCACCGGAATGTATGCCGCCGGCAAAGCA-3′ (forward), and TTTTGCGGCCGCTCAGACGACTAAACTTTCCC-3′ (reverse). PCR with the following primers the former deletion endogenous (isoform 6–2) Ang-1 in T47D cells was generated by PCR amplification with both cDNA and the following primers, 5′-TTTTGAATTCATGGCGCTGTTGGCCGGC-3′ (forward) and 5′-TTTTGCGGCCGCTCAGACGACTAAACTTTCCC-3′ (reverse). Total RNAs were extracted from T47D cells using RNA extraction buffer (38% (w/w) phenol, 0.8 M guanidine thiocyanate, 0.4 M ammonium thiocyanate, 0.1 M sodium acetate, and 5% (v/v) glycerol), and cDNA was prepared from 2 μg of total RNAs with the High-Capacity cDNA Reverse Transcription kit (Thermo Fisher Scientific, Inc.). The resulting cDNA was used for PCR amplification. The resultant cDNAs were subcloned into the EcoRI/NotI restriction sites of the CSII-CMV-MCS-IRES2-Bsd (RIKEN BioResource Center).

Ang-1 full-length (iso1)-GFP (Ang-1/iso1-FL) was generated by PCR amplification of the amino acids 1 to 649 of human Ang-1 with the following primers, 5′-TTTTGCTAGCATGCAACAGGACGGACTTGG-3′ (forward) and 5′-TTTTGAATTCGGACGACTAAACTTTCCCGAC-3′ (reverse). Ang-1/ΔCD was generated by PCR amplification of the amino acids 1 to 298 of human Ang-1 with the following primers, 5′-TTTTGCTAGCATGCAACAGGACGGACTTGG-3′ (forward) and 5′- TTTTGAATTCGGCAGCAGGGGCACCTGACGT-3′ (reverse). Ang-1/CS was generated by inverse PCR with the following primers: 5′-GCAGCAGCTACGTCAGGAGCCCCAGCAGCCCAGACAAGTGCTGCTGCCCCGAGG-3′ (forward) and 5′-TAGTGTGCGGGCTGCTCTGGCTCCAGCTGATGCCCAGGAGGAGGAAGATGAGG-3′ (reverse). Then, Ang-/CS inserted cDNA was generated by PCR amplification in the same way as Ang-1/iso1-FL. The resultant DNAs were subcloned into the NheI/EcoRI restriction sites of the pAcGFP-N1 (Takara Bio Inc.). After that, these GFP-fusion Ang-1 genes were digested with NheI/NotI and subcloned into NheI/NotI restriction sites of the CSII-CMV-MCS-IRES2-Bsd.

### Proliferation assay

The proliferative capacities of T47D parent and Ang-1/KO cells were measured using 3-[4,5-dimethylthiazyol-2yl]-2,5-diphenyltetrazolium bromide assay (Nakalai Tesque, Inc). Cells were seeded in 96-well plates at a concentration of 2000 cells/well containing 200 *μ*l of DMEM cell culture medium supplemented with 10% FBS. The 20 μl of 3-[4,5-dimethylthiazyol-2yl]-2,5-diphenyltetrazolium bromide solution was added every 24 h for 3 days, and cells were incubated for 4 h at 37 °C. Then, 200 μl DMSO were added to each well. The absorbance at 570 nm was measured to quantify the number of living cells (35, 36, 37).

### Animal

T47D parent and Ang-1/KO cells were suspended in 0.3 ml of 2.6 × 10^7^ cells/ml using serum-free DMEM and mixed with 0.5 ml of Corning Matrigel Matrix growth factor reduced (Corning) on ice. Then, 100 μl suspension of both cells were injected subcutaneously into the right and left groin regions of female BALB/c nude mice, 8-week-old, (Jackson Laboratory Japan), respectively. On day 14, the mice were sacrificed, and the tumors were removed. The weight of each tumor was measured and fixed in 4% paraformaldehyde (pH 7.2) and embedded in paraffin. The animal experiments were approved by the Institutional Committee for Experiments of the Institute of Microbial Chemistry.

### PAS/CD31 dual staining

Tissue sections were then processed by deparaffinization with xylene, rehydration through an alcohol gradient, peroxidase clearing with 0.3% H_2_O_2_ methanol, the antigen retrieval was performed by autoclave in citrate buffer (pH 6.0; Nichirei Biosciences, Inc) for 10 min. The blocking was performed using 5% normal goat serum (GIBCO 16210-064). The sections were incubated with anti-CD31 (abcam 182981, dilution 1:1000) at room temperature for 2 h and then treated with the ChemMate ENVISION kit (K5007, Agilent Technologies, Inc., Santa Clara). The chromogenic reaction was performed using 3,3′-diaminobenzidine tetrahydrochloride (Agilent Technologies, Inc). After that, the slides were rinsed with distilled water twice for 1 min, treated with 1% periodic acid solution (40911, Muto Pure Chemicals Co, Ltd) at a room temperature for 5 min, and rinsed again with distilled water for 3 min. The slides were incubated with Schiff solution (40922, Muto Pure Chemicals Co, Ltd) at a room temperature for 15 min, rinsed with distilled water, and finally counterstained with hematoxylin (FUJIFILM Wako Pure Chemical Corporation).

### Reverse transcription-polymerase chain reaction

To check the expression levels of Ang-1 isoforms 1, 2, 3, 4, 5, 6 and 6-2, we performed RT-PCR. The sequences of the primers used to confirm as followed: 5′-CCTCAGGTGTTCCCAGCATT-3′ (forward) and 5′-TCGTGGAGGGAGGTGACTT-3′ (reverse). To examine the expression level of Ang-1/iso1-FL, Ang-1/ΔCD, and Ang-1/CS, we performed RT-PCR. The sequences of the primers used to confirm as followed: 5′-TTTTGCTAGCATGCAACAGGACGGACTTGG-3’ (forward) and 5′-CAGCTCGATCAGGATGGGCACGATGCCGGTG-3′ (reverse).

### Human tissues

Ten breast tumors and matched normal tissue were obtained in Tochigi Cancer Biobank from February to June in 2025. These patients did not receive chemotherapy or radiotherapy before the surgery. Written informed consents were collected from patients enrolled in the study. All experiments were approved and conducted under the supervision of Keio University Bioethics Committee and Tochigi Cancer Center Bioethics Committee. The human subjects in this study abide by the principles of the Declaration of Helsinki. As mRNA extraction from patient samples, we homogenized them with Injection Needle Brown 100 26G (TERUMO CORPORATION), Terumo Syringe 1.0 ml for Vaccination Slip Tip White (TERUMO CORPORATION) and 0.25 ml of RNA extraction buffer. After that, we added 0.75 ml of it to each sample and extracted mRNA derived from patient samples. To identify whether Ang-1 isoform 6-2 expresses in BC patients, we used following primers, P1: 5′- ATGCAACAGGACGGACTTGG-3′ (forward) and 5′-CGGTGATGGTAATCCTCCGG-3′ (reverse), P2: 5′- ATCCAGGTGACCGTGTCCAAC-3′ (forward) and 5′- AAGGACGATGAGCTCTGCGTAG-3′ (reverse), P3: 5′-CCCCACCAGCTATGATTCCC-3′ (forward) and 5′- TCGTGGAGGGAGGTGACTT-3′ (reverse), and P4: 5′- ACAACCCCTACGTTGAGTGC-3′ (forward) and 5′- ACGTCTCCAGGGTATCCTCC-3′ (reverse). To examine the expression of EMT-related genes, we used following primers, E-cadherin: 5′-TGCTCTTGCTGTTTCTTCGG-3′ (forward) and 5′-TGCCCCATTCGTTCAAGTAG-3′ (reverse), vimentin: 5′-GAGATGCTTCAGAGAGAGGAAG-3′ (forward) and 5′-AGAGAGGTCAGCAAACTTGG-3′ (reverse) and N-cadherin: 5′-GGAGAAGAAGACCAGGACTATG-3′ (forward) and 5′-CTCACCACCACTACTTGAGGA-3′ (reverse). To normalize PCR product with GAPDH, we used following primers, GAPDH: 5′-GATTCCACCCATGGCAAATTCC-3′ (forward) and 5′-CACGTTGGCAGTGGGGAC-3′. Band intensities corresponding to mRNA signals were measured using ImageJ (National Institutes of Health, https://imagej.nih.gov/ij/).

## Data availability

All data are contained within the article. Any inquiries regarding the data should be directed to the corresponding author, S.S., simizu@applc.keio.ac.jp.

## Statistical analysis

Differences between two groups were analyzed by the two tailed student’s *t* test. The results were expressed as the means ± SD. *P* values < 0.05 were considered to be statistically significant.

## Supporting information

This article contains supporting information.

## Conflict of interests

The authors declare that they have no conflict of interest with the contents of this article.
