# Supplementary material for: Loss of angulin-1/LSR promotes vasculogenic mimicry and epithelial-mesenchymal transition in breast cancer
Source: J Biol Chem. 2025 Aug 27;301(10):110635. doi: 10.1016/j.jbc.2025.110635 (PMC12493129; doi:10.1016/j.jbc.2025.110635)
Supplement: Supplementary information [file mmc1.docx]

**Loss of Angulin-1/LSR promotes vasculogenic mimicry and epithelial–mesenchymal transition in breast cancer**

**Authors:** Yuma Yoshioka^1^, Chisato Nosaka^2^, Tomokazu Ohishi^2^, Minami Nakajima^1^, Yumiko Ishikawa^4^, Tomoka Toyota^3^, Masaru Takemae^3^, Naoto Kubota^4^, Jumpei Muramatsu^5^, Daisuke Tatsuda^2^, Masuo Kondoh^6,7^, Hiroaki Onoe^5^, Jiro Ando^3^, Manabu Kawada^2^, Hidenori Ojima^4^ and Siro Simizu^1^

1: Department of Applied Chemistry, Faculty of Science and Technology, Keio University, Japan

2: Laboratory of Oncology, Institute of Microbial Chemistry (BIKAKEN), Microbial Chemistry Research Foundation, Japan

3: Department of Breast Surgery, Tochigi Cancer Center, Japan

4: Division of Molecular Pathology, Research Institute, Tochigi Cancer Center, Japan

5: Department of Mechanical Engineering, Faculty of Science and Technology, Keio University, Japan

6: Graduate School of Pharmaceutical Sciences, Osaka University, Japan.

7: Center for Infectious Disease Education and Research (CiDER), Osaka University, Japan

**p.2. Supporting information Fig. S1**

**p.3. Supporting information Fig. S2**

**p.4. Supporting information Fig. S3**

**p.6. Supporting information Fig. S4**

**p.7. Supporting information Fig. S5**

**Supporting information Figure S1**

*A,* Tumor growth curves. Tumor volumes were measured at the indicated time points.

*B,* Quantification of total vessel numbers. VM channels and angiogenesis-derived vessels were counted. Data are presented as means ± SD. **p* < 0.05; N.S., not significant.

**Supporting information Figure S2**

Sequence analysis of endogenous Ang-1 in T47D cells. Isoform 1 and isoform 6-2 were compared using CLUSTALW. Isoform 6-2 lacks amino acid residues 1-48 and 240-308. The region spanning residues 240-308 is replaced with a valine residue (red), resulting in the loss of both the transmembrane (yellow) and cysteine-rich (green) domains. IG-like V domain is colored by blue.

**Supporting information Figure S3**

*A,* Multiple primer sets designed to identify and quantify Ang-1 expression. P1-negative and P2-positive samples were defined as expressing isoform 6-2. Since P3 targets a region conserved among the isoforms registered in UniProt, it yields a positive signal as long as Ang-1 is expressed. P4 was used for quantification of isoform 6-2 (Figure 9). Although the band appears at the same position as isoform 5, quantification was performed specifically for isoform 6-2 by identifying P1-negative and P2-positive patterns. Taken together, samples showing P1-negative, P2-positive and P3-positive results were identified as expressing isoform 6-2 and quantified using P4. Other expression patterns were interpreted as follows: P1-positive, P2-positive, and P3-positive: isoform 2 or one of isoforms 3, 4 or 5 (distinguished by PCR product size); P1-negative, P2-negative and P3-positive: isoform 6; P1-positive, P2-negative and P3-positive: novel isoform; P1-negative, P2-negative and P3-negative: no expression. *B,* Representative images showing that the designed multiple primer sets effectively amplified the target sequences in various Ang-1-re-expressing cell lines. *C,* Representative images demonstrating that the primer sets successfully detected target sequences in 4 BC cell lines. Expression of Ang-1 isoform 6-2 was observed in MCF-7, MDA-MB-231 and T47D cells. *D,* RT-PCR analysis of BC patient tissue samples confirmed the expression of various Ang-1 mRNA isoforms, as well as E-cadherin and Vimentin.

**Supporting information Figure S4**

*A,* Schematic representation of full-length Ang-1 isoform 1 (Ang-1-iso1-FL), a C-terminal cytoplasmic domain-deleted form retaining the CR domain (Ang-1-iso1/ΔCD) and a mutant in which 10 cysteine residues surrounding the CR domain were substituted with serine residues, shown as white “S” (Ang-1-iso1/CS). *B,* Various Ang-1-iso1-GFP-re-expressing MCF-7 cell lines were established, and their expression was confirmed by immunoblotting. mRNA expression levels of Ang-1-iso1-FL, Ang-1-iso1/ΔCD and Ang-1-iso1/CS were also assessed.

*C–E*, Cells expressing Ang-1-iso1-FL, Ang-1-iso1/ΔCD or Ang-1-iso1/CS were seeded on Matrigel-coated 96-well plates (3.2 × 10⁴ cells/well). *C,* Representative images at 24 h after seeding. *D,* Confocal laser scanning microscopy images of representative fields. *E,* Tube numbers were counted in four randomly selected fields (n = 3). Data are presented as means ± SD. ***p* < 0.01. N.D., not detected. Scale bars, 100 µm.

**Supporting information Figure S5**

Survival analysis of BC patients based on Ang-1 expression levels, using data obtained from GEPIA.
